# The Global Plastics Treaty: An Endocrinologist's Assessment

**DOI:** 10.1210/jendso/bvad141

**Published:** 2023-11-14

**Authors:** Marina Olga Fernandez, Leonardo Trasande

**Affiliations:** Laboratorio de Neuroendocrinología, Instituto de Biología y Medicina Experimental (IBYME), Consejo Nacional de Investigaciones Científicas y Técnicas, Vuelta de Obligado 2490, Ciudad Autónoma de Buenos Aires, C1428ADN, Argentina; Department of Pediatrics, NYU Grossman School of Medicine, New York, NY 10016, USA; Department of Population Health, NYU Grossman School of Medicine, New York, NY 10016, USA; NYU Wagner Graduate School of Public Service, New York, NY 10016, USA

**Keywords:** endocrine-disrupting chemicals, international legally binding instrument on plastic pollution, intergovernmental negotiating committee, bisphenol A

## Abstract

Plastics are everywhere. They are in many goods that we use every day. However, they are also a source of pollution. In 2022, at the resumed fifth session of the United Nations Environment Assembly, a historic resolution was adopted with the aim of convening an Intergovernmental Negotiating Committee to develop an international legally binding instrument on plastic pollution, including in the marine environment, with the intention to focus on the entire life cycle of plastics. Plastics, in essence, are composed of chemicals. According to a recent report from the secretariat of the Basel, Rotterdam, and Stockholm conventions, around 13 000 chemicals are associated with plastics and plastic pollution. Many of these chemicals are endocrine-disrupting chemicals and, according to reports by members of the Endocrine Society and others, exposure to some of these chemicals causes enormous costs due to the development of preventable diseases. The global plastics treaty brings the opportunity for harmonized, international regulation of chemicals with endocrine disrupting properties present in plastic products.

## The Problem of Plastic Pollution

Plastics are everywhere. They are a hallmark of modern society, and they are part of many goods that we use every day; they are in food containers, cars, cables, cell phones, and medical devices, among other products. They are also a major source of pollution [1]. Plastic litter ends up leaking into the environment due to poor waste management. However, plastic pollution is more than plastic waste. Plastics pollute throughout their entire life cycle.

## Toward a Legally Binding Instrument to End Plastic Pollution

Recognizing the need to address plastic pollution, in 2022, at the resumed fifth session of the United Nations Environment Assembly (UNEA 5.2), a historic resolution was adopted (UNEA 5/14) [2]. In this resolution, UNEA “requested the Executive Director to convene an intergovernmental negotiating committee (INC), to begin its work during the second half of 2022, with the ambition of completing its work by the end of 2024”. The mandate charged the Committee with developing an international legally binding instrument on plastic pollution, including in the marine environment. The mandate also charged the INC with focusing on the entire life cycle of plastics, from production to consumption to end of life. However, there is temptation to focus only on the visible aspect of plastics pollution (ie, plastic waste or plastic litter).

## Chemicals in Plastics

According to a recent report from the Secretariat to the Basel, Rotterdam, and Stockholm conventions (BRS) [3], around 13 000 chemicals are associated with plastics and plastic pollution. These numbers are an estimate, but we can agree that they are more than was previously thought. From these chemicals, 24% are of potential concern for human and environmental health, and not regulated worldwide, whereas Multilateral Environmental Agreements like the Stockholm Convention, the Minamata Convention or the Montreal Protocol, regulate only 1% of these chemicals globally.

## Chemicals of Concern: Endocrine-Disrupting Chemicals

Many chemicals found in plastics affect the endocrine systems in both wildlife and humans, and are endocrine-disrupting chemicals (EDCs) [4]. An EDC is an exogenous chemical, or a mixture of chemicals, that can interfere with any aspect of hormone action [5]. Increasingly and especially in recent years, the interest of the public health community on EDCs effects has been on the rise, as scientific advances have made it clear that early exposure to these chemicals are influencing physiology, altering development, and increasing disease risk, even at low levels of exposure that are relevant to hormone biology. EDCs found in plastics include bisphenols, phthalates, and UV stabilizers, among other chemicals. Exposure to these chemicals is linked to the development of diseases, and, more importantly, exposure during vulnerable periods of development, like the prenatal, neonatal, or pubertal periods, cause damage that is irreversible, consistent with the concept of “developmental origins of health and disease”. Research shows that disease burden causes enormous costs due to the development of preventable diseases in response to exposure to EDCs [6‐9].

## Regulation of Chemicals in Plastics: The Example of Bisphenol A

Nowadays, agencies around the world regulate chemicals in myriad different ways, sometimes with different approaches to different sectors of the economy, even if the same chemical is found in multiple sectors (eg, clothing and food contact materials). Generally, different countries or regions will determine their own safe level of exposure for different chemicals, and then update the said safe level at different paces and with different underlying legislation and regulatory principles. Taking the example of bisphenol A (BPA), a prototypical EDC that has been studied and used for many years for the manufacture of epoxy resins and polycarbonate plastics, the current safe dose of exposures varies among countries. The current lowest safe exposure dose is the tolerable daily intake set by the European Food Safety Authority, of 0.2 ng/kg bodyweight per day [10], while other nations do not list safe, permitted doses of exposure. In spite of these discrepancies, and recognizing the safety concerns backed by emerging science, many countries banned the commercialization and manufacture of baby bottles containing BPA, recognizing that infants can be exposed directly through polycarbonate bottles. However, this approach does not take into account other ways of exposure (eg, in utero exposure or exposure via breastmilk). Studies in different countries found BPA in the urine of pregnant women [11, 12] and in breastmilk [13, 14], so these can account for dietary exposures in fetuses and infants.

When we regulate chemicals individually, we tend to ban 1 and replace with another. We do substitutions, just changing one chemical that we have evidence of its effect (eg, BPA) for another that we still do not have as much evidence to confirm their effects, as few studies at that time were performed by independent researchers (eg, bisphenol S or F). By doing these substitutions, BPA-free goods could incorporate other types of bisphenols with similar hazards, resulting in a “regrettable substitution” [15].

## The Need for the Global Treaty to Regulate Chemicals in Plastics

Considering these issues, it is clear that there exists a pressing need for a global treaty that will address hazardous chemicals in plastics in a harmonized way. This approach will help address other important things like waste management and circularity, but the treaty must incorporate public health as a critical aim. Toxic chemicals do not belong in a circular economy, as recycling alone will continue to circulate chemicals that are unsafe for human and environmental health.

## Data Availability

Data sharing is not applicable to this article as no datasets were generated or analyzed during the current study

## Abbreviations

BPA: bisphenol A
BRS: Basel, Rotterdam, and Stockholm
EDC: endocrine-disrupting chemical
INC: intergovernmental negotiating committee
UNEA: United Nations Environment Assembly
UV: ultraviolet
